# Supplementary material for: Tourism and the Global Vectoring of Antimicrobial-Resistant Disease: What Countries Are Most Impacted?
Source: Antibiotics (Basel). 2025 Oct 22;14(11):1055. doi: 10.3390/antibiotics14111055 (PMC12649158; doi:10.3390/antibiotics14111055)
Supplement: Supplementary file 1 [file antibiotics-14-01055-s001.zip › antibiotics-3866464-supplementary.pdf]

Supplementary Materials

Tourism and the Global Vectoring of Antimicrobial-Resistant Disease: What Countries Are Most Impacted?

Methods

Application of the gravity model

The model was first introduced by Zipf in 1946 to study inter-city travel in the USA (1), and later it was applied to global trade flows in 1962 by Tinbergen (2), and further theoretical economic foundations were developed in 1979 and 2010 by Anderson (3,4). Wilson, in 1967, motivated the model as the entropy-maximizing solution of a statistical mechanics formulation of intra-urban trip distribution patterns (5). The model saw a resurgence in tourism research in the 2000s, with Nadal and Gallego providing an extensive survey of recent research in 2022 (6-10).

Implementation of the gravity model can be explained by the using simple equations. For travellers departing from country i the attraction to destination country j is measured as:

$$Attraction_{ij} = \frac{(Total\ Outbound\ Travel\ From\ i) * (Total\ Inbound\ Travel\ to\ j)}{(Distance\ Between\ i\ and\ j)^{\gamma}}$$

Equation (S1)

The parameter γ determines the sensitivity of travel to distance. The number of travellers from i going to j is then estimated allocating departures proportionally according to relative attractiveness of destination j versus all other possible destinations:

$$Travellers_{ij} = K.\frac{Attraction_{ij}}{\sum_j Attraction_{ij}}.(Total\ Outbound\ Travel\ From\ i).$$

Equation (S2)

K is a scaling factor that must account for the fact that persons departing from i may visit more than one destination. In our analysis K=1.34.

The size of the origin and destination pair is determined by their ability to generate or attract travellers, measured by the annual number of tourists departing from and arriving at each destination. While 'distance' can encompass various factors like language connectivity, travel costs, and ease of crossing borders, physical distance is the most used metric. According to Nadal and Gallego, distance is included in 88% of the relevant research papers they surveyed, while travel costs are considered in only 7%. Analysis uses $\gamma = 1$.

The use of Logarithmic responses.

Logarithmic responses to treatments are commonly found in medicine to model the relationships e.g. between drug concentrations and biological response. For small values of the impacts measured in equations (1), (2), and (3), the logarithmic values change by nearly by the

same amount, however, the logarithmic rate of response becomes more muted as the values rise. For the sake of clarity, Supplementary Figure S? shows a chart of the relationship between (EIT+EIV) and (ln(1+EIT+EIV) that we subsequently calculate.

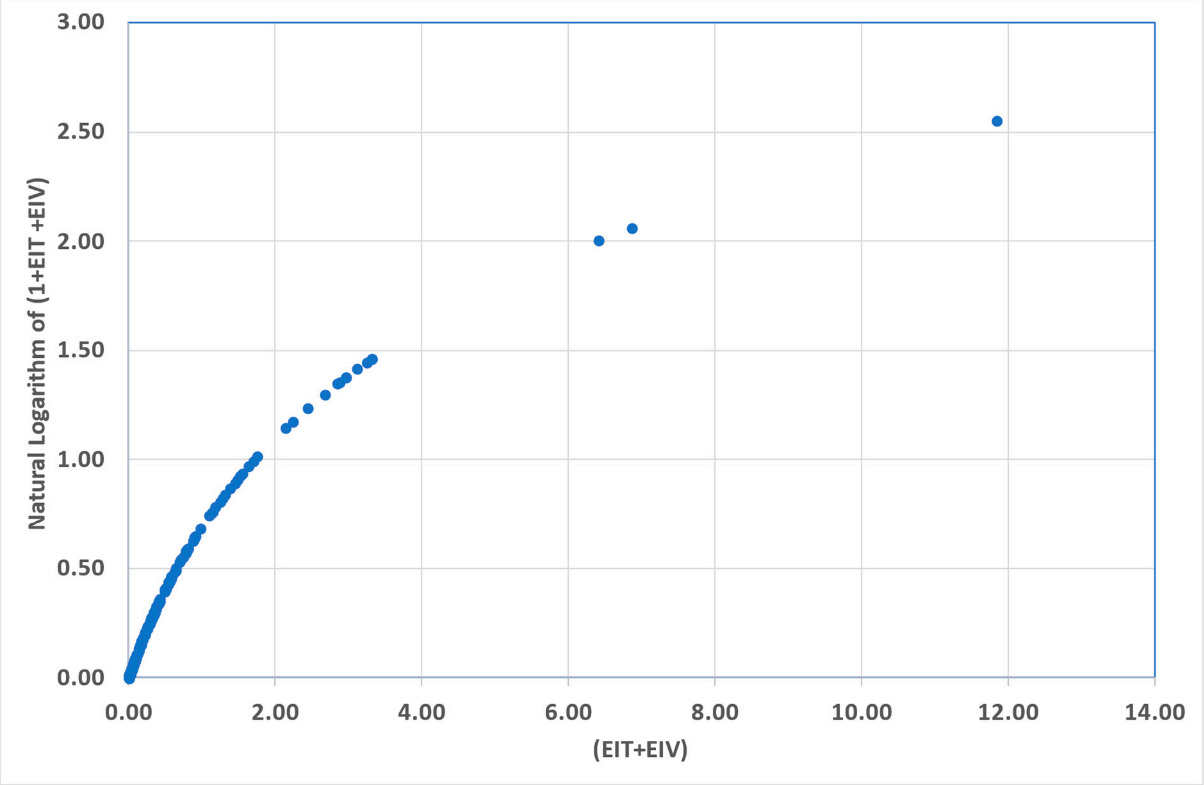

Figure S1. AMR Infection Burden of Travel Combined Impact of Returning Resident Travellers and Arriving Visitors (EIT + EIV) plotted against its logarithmically transformed value for all 241 countries.

Supplementary Figure S2

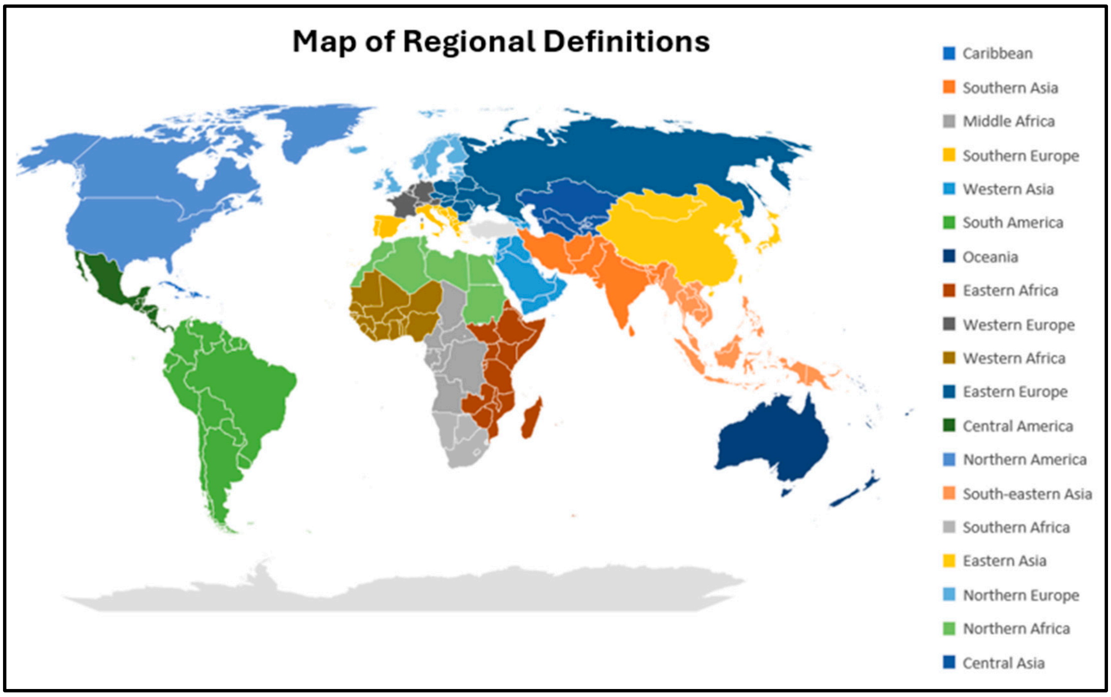

Results

Supplementary Figure S3: Departures Per Capita versus Per Capita GDP

(for countries with departures data)

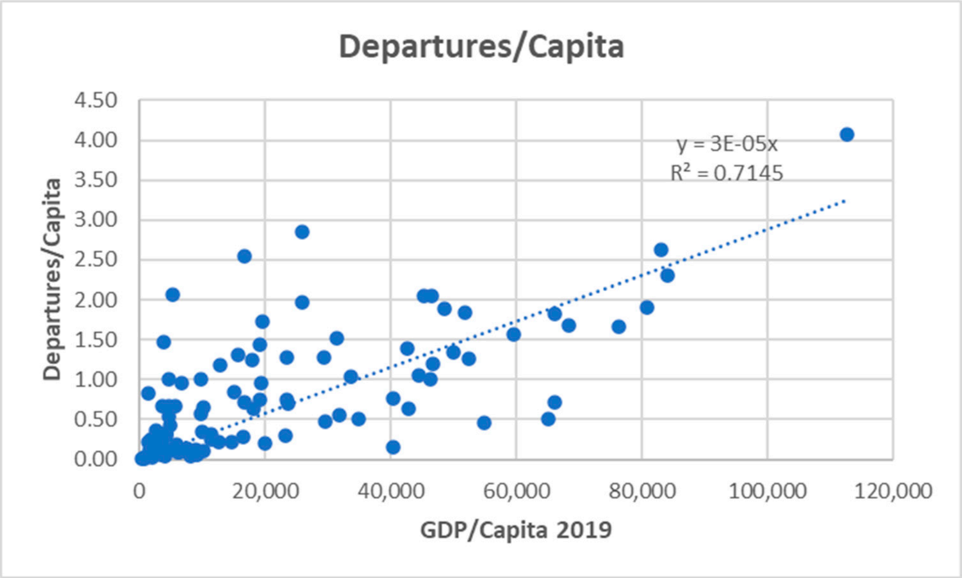

Supplementary Table S1: Estimated Inter-Regional Annual Trips 2019:

Rows Showing Origin and Columns Showing Destination

	Caribbean	Central America	Central Asia	Eastern Africa	Eastern Asia	Eastern Europe	Middle Africa	Northern Africa	Northern America	Northern Europe	Oceania	South America	South-eastern Asia	Southern Africa	Southern Asia	Southern Europe	Western Africa	Western Asia	Western Europe
**Caribbean**	1,755	8,007	287	254	3,661	1,522	131	272	16,401	1,375	397	7,008	703	294	617	1,147	485	1,088	1,575
**Central America**	1,587	8,723	937	723	13,887	4,520	354	747	43,688	3,921	1,662	15,574	2,615	838	2,016	3,147	1,248	3,250	4,474
**Central Asia**	62	971	1,703	266	6,992	2,440	92	292	2,193	1,281	281	1,066	1,282	221	2,620	1,066	291	2,648	1,548
**Eastern Africa**	64	856	291	1,238	3,123	1,197	268	291	1,798	785	304	1,587	909	1,465	824	843	542	1,737	1,002
**Eastern Asia**	655	12,010	5,774	2,186	161,606	13,684	754	1,879	23,706	8,886	4,914	10,517	28,032	2,080	14,942	7,143	2,307	13,253	10,456
**Eastern Europe**	572	7,914	3,657	1,804	25,954	55,403	790	3,819	19,947	29,372	1,432	9,310	5,073	1,533	6,589	27,422	2,908	16,132	59,072
**Middle Africa**	13	166	42	107	424	215	155	55	357	153	40	322	110	108	106	174	252	238	197
**Northern Africa**	157	1,988	685	787	5,627	4,902	314	1,065	4,899	3,545	374	2,843	1,266	545	1,573	5,855	1,162	5,227	4,888
**Northern America**	2,818	38,008	1,832	1,319	23,715	9,722	655	1,580	50,820	9,056	2,098	21,589	4,352	1,425	3,805	6,946	2,433	6,455	10,118
**Northern Europe**	309	4,205	1,400	738	11,396	18,647	341	1,499	11,231	16,246	644	4,689	2,165	663	2,544	9,212	1,300	5,397	31,029
**Oceania**	74	1,521	239	206	5,519	761	72	124	2,217	529	995	1,675	1,485	249	666	453	201	794	629
**South America**	555	5,945	334	419	4,193	1,692	214	329	8,808	1,422	698	15,465	999	553	785	1,289	703	1,407	1,681
**South-eastern Asia**	269	4,745	2,296	1,375	63,029	5,738	430	912	9,209	3,655	3,003	5,408	30,679	1,350	8,845	3,209	1,215	6,962	4,421
**Southern Africa**	77	1,021	254	1,239	2,989	1,075	298	240	2,013	749	358	2,130	878	1,946	704	779	555	1,308	941
**Southern Asia**	83	1,264	1,496	469	9,876	2,778	147	435	2,789	1,507	459	1,552	2,474	382	3,449	1,405	439	4,763	1,883
**Southern Europe**	1,090	13,861	4,261	3,101	35,218	53,533	1,537	9,983	35,828	38,599	2,172	17,961	7,262	2,722	8,673	43,050	6,242	22,251	61,044
**Western Africa**	68	789	161	257	1,596	942	263	246	1,797	738	139	1,508	381	258	382	849	917	856	937
**Western Asia**	496	6,929	4,827	3,473	31,157	24,361	1,053	4,981	16,088	11,100	1,843	9,362	7,231	2,265	12,413	13,176	3,295	44,617	15,097
**Western Europe**	742	9,777	3,015	1,867	25,157	43,388	895	4,565	25,885	74,386	1,481	11,601	4,956	1,671	5,802	32,884	3,513	13,165	67,649

Supplementary Table S2: Country level data on AMR Resistance Rates, Prevalence of Infectious Disease Colonization proxy, and Estimated Burden of Travel

ISO3	Country	Region	3rd Generation Cephalosporin Resistance Rate for E.coli (missing data filled with regional average)	Prevalence of Infectious Disease Colonization(Normalised Infection Death Rate)	Burden of Travel LN(1+ERT+ERV)
ABW	ARUBA	Caribbean	29%	95.16	0.84
AFG	AFGHANISTAN	Southern Asia	46%	103.01	0.00
AGO	ANGOLA	Middle Africa	34%	111.59	0.01
AIA	ANGUILLA	Caribbean	29%	95.16	0.58
ALB	ALBANIA	Southern Europe	18%	90.83	0.46
AND	ANDORRA	Southern Europe	18%	91.98	2.55
ANT	NETHERLANDS ANTILLES	Caribbean	29%	95.16	0.09
ARE	UNITED ARAB EMIRATES	Western Asia	26%	90.03	0.44
ARG	ARGENTINA	South America	4%	98.03	0.28
ARM	ARMENIA	Western Asia	33%	91.10	0.11
ASM	AMERICAN SAMOA	Oceania	8%	93.73	0.40
ATF	FRENCH SOUTHERN TERRITORIES	Eastern Africa	41%	113.04	0.00
ATG	ANTIGUA AND BARBUDA	Caribbean	29%	92.67	0.64
AUS	AUSTRALIA	Oceania	8%	90.72	0.27
AUT	AUSTRIA	Western Europe	10%	90.46	0.68
AZE	AZERBAIJAN	Western Asia	33%	92.52	0.08
BDI	BURUNDI	Eastern Africa	8%	125.00	0.01
BEL	BELGIUM	Western Europe	9%	95.86	0.41
BEN	BENIN	Western Africa	33%	130.18	0.00
BFA	BURKINA FASO	Western Africa	40%	143.74	0.00
BGD	BANGLADESH	Southern Asia	57%	97.33	0.00
BGR	BULGARIA	Eastern Europe	18%	92.11	0.31
BHR	BAHRAIN	Western Asia	43%	89.51	0.48
BHS	BAHAMAS	Caribbean	29%	91.72	0.87
BIH	BOSNIA AND HERZEGOVINA	Southern Europe	8%	90.56	0.14
BLM	ST. BARTHÉLEMY	Caribbean	29%	95.16	0.09
BLR	BELARUS	Eastern Europe	24%	90.59	0.17
BLZ	BELIZE	Central America	29%	92.67	0.32
BMU	BERMUDA	Northern America	16%	91.82	1.18
BOL	BOLIVIA	South America	22%	98.14	0.02
BRA	BRAZIL	South America	23%	94.67	0.04
BRB	BARBADOS	Caribbean	29%	96.01	0.27
BRN	BRUNEI	South-eastern Asia	10%	91.69	1.38
BTN	BHUTAN	Southern Asia	28%	96.64	0.05
BVT	BOUVET ISLAND	South America	22%	94.48	0.04
BWA	BOTSWANA	Southern Africa	13%	103.06	0.09
CAF	CENTRAL AFRICAN REPUBLIC	Middle Africa	28%	153.08	0.00
CAN	CANADA	Northern America	12%	92.00	0.32
CCK	COCOS (KEELING) ISLANDS	Oceania	8%	91.42	0.27
CHE	SWITZERLAND	Western Europe	10%	91.73	0.57

CHL	CHILE	South America	16%	92.06	0.07
CHN	CHINA	Eastern Asia	36%	90.54	0.02
CIV	CÔTE D'IVOIRE	Western Africa	45%	122.24	0.01
CMR	CAMEROON	Middle Africa	35%	125.87	0.01
COD	CONGO (DRC)	Middle Africa	35%	129.46	0.00
COG	CONGO (REPUBLIC)	Middle Africa	34%	202.86	0.00
COK	COOK ISLANDS	Oceania	8%	95.41	1.38
COL	COLOMBIA	South America	25%	91.18	0.02
COM	COMOROS	Eastern Africa	41%	110.56	0.01
CPV	CAPE VERDE	Western Africa	33%	97.60	0.09
CRI	COSTA RICA	Central America	29%	90.68	0.08
CUB	CUBA	Caribbean	33%	95.86	0.03
CUW	CURAÇAO	Caribbean	29%	95.16	0.44
CXR	CHRISTMAS ISLAND	Oceania	8%	91.42	0.29
CYM	CAYMAN ISLANDS	Caribbean	29%	95.16	1.36
CYP	CYPRUS	Western Asia	30%	90.42	0.35
CZE	CZECH REPUBLIC	Eastern Europe	12%	92.51	0.47
DEU	GERMANY	Western Europe	11%	92.75	0.29
DJI	DJIBOUTI	Eastern Africa	41%	109.41	0.02
DMA	DOMINICA	Caribbean	29%	95.16	0.30
DNK	DENMARK	Northern Europe	7%	94.22	0.97
DOM	DOMINICAN REPUBLIC	Caribbean	27%	88.58	0.06
DZA	ALGERIA	Northern Africa	43%	90.87	0.01
ECU	ECUADOR	South America	20%	93.24	0.03
EGY	EGYPT	Northern Africa	43%	92.59	0.01
ERI	ERITREA	Eastern Africa	41%	134.71	0.00
ESH	WESTERN SAHARA	Northern Africa	31%	92.72	0.02
ESP	SPAIN	Southern Europe	16%	92.37	0.35
EST	ESTONIA	Northern Europe	7%	90.77	0.91
ETH	ETHIOPIA	Eastern Africa	54%	107.79	0.00
FIN	FINLAND	Northern Europe	6%	90.53	0.65
FJI	FIJI	Oceania	8%	94.72	0.30
FLK	FALKLAND ISLANDS (ISLAS MALVINAS)	South America	22%	94.48	0.04
FRA	FRANCE	Western Europe	9%	93.21	0.58
FRO	FAROE ISLANDS	Northern Europe	9%	94.59	0.49
FSM	MICRONESIA	Oceania	8%	95.49	0.08
GAB	GABON	Middle Africa	34%	101.89	0.02
GBR	UNITED KINGDOM	Northern Europe	9%	95.74	0.35
GEO	GEORGIA	Western Asia	24%	91.60	0.30
GGY	GUERNSEY	Western Europe	10%	92.97	0.41
GHA	GHANA	Western Africa	42%	114.07	0.01
GIB	GIBRALTAR	Southern Europe	18%	92.30	0.35
GIN	GUINEA	Western Africa	33%	136.16	0.00
GLP	GUADELOUPE	Caribbean	29%	95.16	0.09
GMB	GAMBIA	Western Africa	33%	107.16	0.02
GNB	GUINEA-BISSAU	Western Africa	33%	121.99	0.00
GNQ	EQUATORIAL GUINEA	Middle Africa	34%	102.24	0.02
GRC	GREECE	Southern Europe	16%	94.89	0.45
GRD	GRENADA	Caribbean	29%	93.18	0.30
GRL	GREENLAND	Northern America	16%	92.43	0.15
GTM	GUATEMALA	Central America	27%	97.65	0.03
GUF	FRENCH GUIANA	South America	22%	94.48	0.04
GUM	GUAM	Oceania	8%	92.06	1.46
GUY	GUYANA	South America	22%	94.84	0.06

HKG	HONG KONG	Eastern Asia	12%	91.41	2.06
HND	HONDURAS	Central America	27%	93.18	0.03
HRV	CROATIA	Southern Europe	9%	90.43	1.46
HTI	HAITI	Caribbean	29%	105.12	0.01
HUN	HUNGARY	Eastern Europe	15%	90.50	0.78
IDN	INDONESIA	South-eastern Asia	48%	98.52	0.01
IMN	ISLE OF MAN	Northern Europe	9%	94.59	0.43
IND	INDIA	Southern Asia	46%	104.48	0.00
IRL	IRELAND	Northern Europe	11%	92.56	0.53
IRN	IRAN	Southern Asia	41%	90.51	0.01
IRQ	IRAQ	Western Asia	45%	90.75	0.05
ISL	ICELAND	Northern Europe	7%	91.93	1.24
ISR	ISRAEL	Western Asia	7%	91.52	0.59
ITA	ITALY	Southern Europe	22%	91.36	0.23
JAM	JAMAICA	Caribbean	29%	91.12	0.13
JEY	JERSEY	Western Europe	10%	92.97	0.41
JOR	JORDAN	Western Asia	25%	90.16	0.06
JPN	JAPAN	Eastern Asia	18%	99.53	0.06
KAZ	KAZAKHSTAN	Central Asia	35%	91.75	0.08
KEN	KENYA	Eastern Africa	64%	108.37	0.00
KGZ	KYRGYZSTAN	Central Asia	35%	91.12	0.17
KHM	CAMBODIA	South-eastern Asia	47%	103.63	0.03
KIR	KIRIBATI	Oceania	8%	106.68	0.04
KNA	ST. KITTS AND NEVIS	Caribbean	29%	94.69	1.01
KOR	SOUTH KOREA	Eastern Asia	26%	92.79	0.11
KWT	KUWAIT	Western Asia	32%	90.31	0.24
LAO	LAOS	South-eastern Asia	24%	101.13	0.11
LBN	LEBANON	Western Asia	32%	92.49	0.05
LBR	LIBERIA	Western Africa	20%	118.54	0.01
LBY	LIBYA	Northern Africa	30%	90.73	0.04
LCA	ST. LUCIA	Caribbean	29%	93.05	0.43
LIE	LIECHTENSTEIN	Western Europe	10%	92.97	0.89
LKA	SRI LANKA	Southern Asia	45%	93.29	0.01
LSO	LESOTHO	Southern Africa	5%	120.40	0.17
LTU	LITHUANIA	Northern Europe	9%	91.45	0.64
LUX	LUXEMBOURG	Western Europe	11%	91.70	0.63
LVA	LATVIA	Northern Europe	14%	91.15	0.59
MAC	MACAU	Eastern Asia	34%	91.41	1.35
MAF	ST. MARTIN	Caribbean	29%	95.16	0.09
MAR	MOROCCO	Northern Africa	16%	92.99	0.06
MCO	MONACO	Western Europe	10%	96.04	1.45
MDA	MOLDOVA	Eastern Europe	31%	92.54	0.01
MDG	MADAGASCAR	Eastern Africa	18%	115.81	0.00
MDV	MALDIVES	Southern Asia	32%	90.75	0.28
MEX	MEXICO	Central America	29%	91.56	0.11
MHL	MARSHALL ISLANDS	Oceania	8%	99.14	0.09
MKD	MACEDONIA (FYROM)	Southern Europe	35%	89.95	0.03
MLI	MALI	Western Africa	22%	136.61	0.00
MLT	MALTA	Southern Europe	15%	92.81	0.82
MMR	MYANMAR (BURMA)	South-eastern Asia	51%	101.22	0.01
MNE	MONTENEGRO	Southern Europe	18%	89.95	0.41
MNG	MONGOLIA	Eastern Asia	43%	92.38	0.02
MNP	NORTHERN MARIANA ISLANDS	Oceania	8%	92.21	1.42
MOZ	MOZAMBIQUE	Eastern Africa	15%	118.71	0.01
MRT	MAURITANIA	Western Africa	33%	107.30	0.01

MSR	MONTSERRAT	Caribbean	29%	95.16	0.28
MTQ	MARTINIQUE	Caribbean	29%	95.16	0.18
MUS	MAURITIUS	Eastern Africa	26%	91.68	0.13
MWI	MALAWI	Eastern Africa	29%	113.21	0.01
MYS	MALAYSIA	South-eastern Asia	17%	97.87	0.21
MYT	MAYOTTE	Eastern Africa	41%	113.04	0.00
NAM	NAMIBIA	Southern Africa	13%	104.80	0.13
NCL	NEW CALEDONIA	Oceania	8%	91.42	0.50
NER	NIGER	Western Africa	33%	153.23	0.00
NFK	NORFOLK ISLAND	Oceania	8%	91.42	0.23
NGA	NIGERIA	Western Africa	31%	135.04	0.01
NIC	NICARAGUA	Central America	30%	91.12	0.03
NIU	NIUE	Oceania	8%	95.07	0.93
NLD	NETHERLANDS	Western Europe	7%	93.16	0.46
NOR	NORWAY	Northern Europe	5%	93.90	0.76
NPL	NEPAL	Southern Asia	44%	100.25	0.00
NRU	NAURU	Oceania	8%	93.54	0.21
NZL	NEW ZEALAND	Oceania	3%	90.85	0.81
OMN	OMAN	Western Asia	31%	90.52	0.21
PAK	PAKISTAN	Southern Asia	38%	105.77	0.00
PAN	PANAMA	Central America	11%	91.95	0.19
PER	PERU	South America	43%	96.04	0.01
PHL	PHILIPPINES	South-eastern Asia	27%	100.12	0.02
PLW	PALAU	Oceania	8%	101.30	0.94
PNG	PAPUA NEW GUINEA	South-eastern Asia	12%	107.93	0.02
POL	POLAND	Eastern Europe	14%	92.20	0.41
PRI	PUERTO RICO	Caribbean	29%	93.47	0.20
PRK	NORTH KOREA	Eastern Asia	34%	93.42	0.03
PRT	PORTUGAL	Southern Europe	13%	97.77	0.28
PRY	PARAGUAY	South America	8%	92.28	0.34
PSE	PALESTINE	Western Asia	23%	90.14	0.10
PYF	FRENCH POLYNESIA	Oceania	8%	91.42	0.30
QAT	QATAR	Western Asia	22%	89.04	0.36
REU	RÉUNION	Eastern Africa	41%	113.04	0.03
ROU	ROMANIA	Eastern Europe	24%	92.74	0.17
RUS	RUSSIA	Eastern Europe	27%	91.93	0.04
RWA	RWANDA	Eastern Africa	26%	106.83	0.01
SAU	SAUDI ARABIA	Western Asia	28%	90.72	0.14
SDN	SUDAN	Northern Africa	43%	95.44	0.01
SEN	SENEGAL	Western Africa	33%	111.38	0.01
SGP	SINGAPORE	South-eastern Asia	22%	95.02	0.54
SGS	SOUTH GEORGIA AND THE SOUTH SANDWICH ISLANDS	South America	22%	94.48	0.04
SJM	SVALBARD AND JAN MAYEN	Northern Europe	9%	94.59	0.55
SLB	SOLOMON ISLANDS	Oceania	8%	107.24	0.03
SLE	SIERRA LEONE	Western Africa	33%	136.52	0.00
SLV	EL SALVADOR	Central America	29%	93.72	0.07
SMR	SAN MARINO	Southern Europe	18%	92.09	2.00
SOM	SOMALIA	Eastern Africa	41%	153.07	0.00
SPM	SAINT PIERRE AND MIQUELON	Northern America	16%	91.98	0.15
SRB	SERBIA	Southern Europe	12%	91.45	0.08
SSD	SOUTH SUDAN	Eastern Africa	41%	125.13	0.00
STP	SÃO TOMÉ AND PRÍNCIPE	Middle Africa	34%	99.07	0.02
SUR	SURINAME	South America	22%	93.66	0.04
SVK	SLOVAKIA	Eastern Europe	24%	92.38	0.27

SVN	SLOVENIA	Southern Europe	10%	92.25	0.76
SWE	SWEDEN	Northern Europe	5%	92.89	0.74
SWZ	SWAZILAND Eswatini	Southern Africa	13%	108.85	0.40
SYC	SEYCHELLES	Eastern Africa	41%	99.39	0.26
SYR	SYRIA	Western Asia	26%	90.39	0.02
TCA	TURKS AND CAICOS ISLANDS	Caribbean	29%	95.16	1.30
TCD	CHAD	Middle Africa	34%	152.16	0.00
TGO	TOGO	Western Africa	33%	119.11	0.01
THA	THAILAND	South-eastern Asia	32%	96.53	0.06
TJK	TAJIKISTAN	Central Asia	35%	94.99	0.01
TKL	TOKELAU	Oceania	8%	92.80	0.23
TKM	TURKMENISTAN	Central Asia	35%	92.18	0.05
TLS	TIMOR-LESTE	South-eastern Asia	29%	100.72	0.01
TON	TONGA	Oceania	8%	96.39	0.25
TTO	TRINIDAD AND TOBAGO	Caribbean	12%	91.32	0.17
TUN	TUNISIA	Northern Africa	16%	90.97	0.14
TUR	TURKEY	Western Asia	36%	91.00	0.05
TUV	TUVALU	Oceania	8%	97.00	0.22
TWN	TAIWAN	Eastern Asia	15%	95.07	0.20
TZA	TANZANIA	Eastern Africa	38%	109.67	0.00
UGA	UGANDA	Eastern Africa	38%	112.07	0.00
UKR	UKRAINE	Eastern Europe	28%	92.14	0.08
URY	URUGUAY	South America	5%	94.57	0.46
USA	UNITED STATES	Northern America	16%	91.97	0.15
UZB	UZBEKISTAN	Central Asia	35%	93.47	0.04
VCT	SAINT VINCENT AND THE GRENADINES	Caribbean	29%	94.19	0.27
VEN	VENEZUELA	South America	17%	92.88	0.06
VGB	BRITISH VIRGIN ISLANDS	Caribbean	29%	95.16	1.15
VIR	U.S. VIRGIN ISLANDS	Caribbean	29%	92.14	0.99
VNM	VIETNAM	South-eastern Asia	21%	94.36	0.04
VUT	VANUATU	Oceania	8%	98.96	0.23
WLF	WALLIS AND FUTUNA	Oceania	8%	91.42	0.23
WSM	SAMOA	Oceania	8%	94.52	0.30
XXK	KOSOVO	Southern Europe	23%	92.30	0.27
YEM	YEMEN	Western Asia	33%	97.88	0.06
ZAF	SOUTH AFRICA	Southern Africa	13%	102.61	0.08
ZMB	ZAMBIA	Eastern Africa	41%	109.78	0.01
ZWE	ZIMBABWE	Eastern Africa	33%	114.17	0.03

Supplementary Table S3: Country Data for Geolocation and Traveller Numbers

Country	Capital City	Capital City Latitude Degrees	Capital City Longitude Degrees	Population '000 Year 2020	Inbound Non-resident Travellers '000	Outbound Resident Travellers '000
ARUBA	Oranjestad	12.51	-70.01	108	195	3
AFGHANISTAN	Kabul	34.58	69.24	38,326	74	70
ANGOLA	Luanda	-8.84	**13.29**	32,899	22	288
ANGUILLA	The Valley	18.21	**-63.06**	15	17	0
ALBANIA	Tirana	41.33	19.82	2,872	641	831
ANDORRA	Andorra la Vella	42.51	1.52	77	824	14
NETHERLANDS ANTILLES	Willemstad	12.11	-68.93	23	2	1
UNITED ARAB EMIRATES	Abu Dhabi	24.30	54.70	9,248	2,528	2,531
ARGENTINA	Buenos Aires	-34.60	-58.38	44,908	1,113	2,102
ARMENIA	Yerevan	40.18	44.50	2,812	189	258
AMERICAN SAMOA	Pago Pago	-14.28	-170.70	47	6	3
FRENCH SOUTHERN TERRITORIES	Saint-Pierre	-21.34	55.48	1	0.004	0
ANTIGUA AND BARBUDA	St. John's	17.13	-61.85	92	104	8
AUSTRALIA	Canberra	-35.28	149.13	25,544	947	1,647
AUSTRIA	Vienna	48.21	16.37	8,901	3,188	1,564
AZERBAIJAN	Baku	40.41	49.87	10,274	317	754
BURUNDI	Bujumbura	-3.36	29.36	12,054	52	11
BELGIUM	Brussels	50.85	4.35	11,541	934	2,862
BENIN	Porto-Novo	6.50	2.63	12,467	34	57
BURKINA FASO	Ouagadougou	12.37	-1.52	21,232	14	66
BANGLADESH	Dhaka	23.81	90.41	166,427	32	1,400
BULGARIA	Sofia	42.70	23.32	7,020	1,255	923
BAHRAIN	Manama	26.23	50.59	1,494	1,106	382
BAHAMAS	Nassau	25.05	-77.36	406	725	57
BOSNIA AND HERZEGOVINA	Sarajevo	43.86	18.41	3,341	120	82
ST. BARTHÉLEMY	Gustavia	17.90	-62.85	11	1	0
BELARUS	Minsk	53.90	27.56	9,661	1,183	1,033
BELIZE	Belmopan	17.25	-88.76	392	167	11
BERMUDA	Hamilton	32.29	-64.78	64	81	31
BOLIVIA	La Paz	-16.49	-68.12	11,862	124	159
BRAZIL	Brasília	-15.79	-47.88	212,565	635	8,045
BARBADOS	Bridgetown	13.11	-59.60	280	97	23
BRUNEI	Bandar Seri Begawan	4.90	114.94	440	445	55
BHUTAN	Thimphu	27.47	89.64	770	32	9
BOUVET ISLAND	Bouvet Island	-54.43	3.38	1	0	0
BOTSWANA	Gaborone	-24.63	25.92	2,524	74	78
CENTRAL AFRICAN REPUBLIC	Bangui	4.39	18.56	5,272	9	9
CANADA	Ottawa	45.42	-75.70	37,758	3,243	5,592
COCOS (KEELING) ISLANDS	West Island	-12.19	96.83	1	0	0
SWITZERLAND	Bern	46.95	7.45	8,606	1,182	2,652

CHILE	Santiago	-33.45	-70.67	19,207	543	558
CHINA	Beijing	39.90	116.41	1,423,998	16,254	18,541
CÔTE D'IVOIRE	Yamoussoukro	6.83	-5.29	26,478	207	241
CAMEROON	Yaoundé	3.85	11.50	26,137	102	158
CONGO (DRC)	Kinshasa	-4.44	15.27	91,332	163	209
CONGO (REPUBLIC)	Brazzaville	-4.26	15.24	5,635	10	52
COOK ISLANDS	Avarua	-21.21	-159.78	17	17	2
COLOMBIA	Bogotá	4.71	-74.07	50,617	453	668
COMOROS	Moroni	-11.72	43.25	798	5	5
CAPE VERDE	Praia	14.93	-23.51	580	76	9
COSTA RICA	San José	9.93	-84.09	5,105	337	178
CUBA	Havana	23.05	-82.35	11,309	428	101
CURAÇAO	Willemstad	12.12	-68.88	189	129	16
CHRISTMAS ISLAND	Flying Fish Cove	-10.42	105.68	2	0	0
CAYMAN ISLANDS	George Town	19.29	-81.37	67	233	27
CYPRUS	Nicosia	35.19	33.38	1,234	412	229
CZECH REPUBLIC	Prague	50.08	14.44	10,538	3,720	789
GERMANY	Berlin	52.52	13.40	83,268	3,956	13,521
DJIBOUTI	Djibouti	11.57	43.15	1,082	17	14
DOMINICA	Roseau	15.31	-61.38	72	32	3
DENMARK	Copenhagen	55.68	12.57	5,811	3,210	903
DOMINICAN REPUBLIC	Santo Domingo	18.49	-69.93	10,939	755	82
ALGERIA	Algiers	36.75	3.04	43,091	237	885
ECUADOR	Quito	-0.18	-78.47	17,487	211	224
EGYPT	Cairo	30.04	31.24	106,539	1,303	1,313
ERITREA	Asmara	15.32	38.93	3,524	15	18
WESTERN SAHARA	El Aaiún	27.13	-13.16	593	11	8
SPAIN	Madrid	40.42	-3.70	47,330	12,617	2,966
ESTONIA	Tallinn	59.44	24.75	1,329	610	173
ETHIOPIA	Addis Ababa	8.98	38.76	115,638	81	419
FINLAND	Helsinki	60.17	24.94	5,525	329	1,209
FIJI	Suva	-18.12	178.45	918	97	25
FALKLAND ISLANDS (ISLAS MALVINAS)	Stanley	-51.70	-57.85	4	0	0
FRANCE	Paris	48.86	2.35	64,458	21,788	4,545
FAROE ISLANDS	Tórshavn	62.01	-6.79	53	6	10
MICRONESIA	Palikir	6.91	158.16	112	2	2
GABON	Libreville	0.42	9.47	2,268	4	68
UNITED KINGDOM	London	51.51	-0.13	66,951	4,086	13,295
GEORGIA	Tbilisi	41.72	44.83	3,769	773	500
GUERNSEY	St. Peter Port	49.46	-2.54	64	11	9
GHANA	Accra	5.60	-0.19	31,849	113	277
GIBRALTAR	Gibraltar	36.14	-5.35	39	9	5
GUINEA	Conakry	9.64	-13.58	13,042	42	55
GUADELOUPE	Basse-Terre	16.01	-61.71	395	42	10
GAMBIA	Banjul	13.45	-16.58	2,541	62	7
GUINEA-BISSAU	Bissau	11.88	-15.62	1,993	5	6
EQUATORIAL GUINEA	Malabo	3.75	8.74	1,578	3	45
GREECE	Athens	37.98	23.73	10,543	3,401	1,054
GRENADA	St. George's	12.06	-61.75	123	53	5
GREENLAND	Nuuk	64.18	-51.69	56	3	4
GUATEMALA	Guatemala City	14.63	-90.51	17,231	256	274
FRENCH GUIANA	Cayenne	4.92	-52.31	288	3	9
GUAM	Hagåtña	13.47	144.75	169	167	25
GUYANA	Georgetown	6.80	-58.16	792	32	21

HONG KONG	Hong Kong	22.40	114.11	7,502	5,591	15,691
HONDURAS	Tegucigalpa	14.07	-87.19	10,042	232	115
CROATIA	Zagreb	45.82	15.98	4,115	6,002	429
HAITI	Port-au-Prince	18.59	-72.31	11,235	94	72
HUNGARY	Budapest	47.50	19.04	9,770	6,140	3,225
INDONESIA	Jakarta	-6.21	106.85	270,826	1,611	1,742
ISLE OF MAN	Douglas	54.15	-4.49	84	10	14
INDIA	New Delhi	28.61	77.21	1,389,966	1,791	3,566
IRELAND	Dublin	53.35	-6.26	4,925	1,095	1,075
IRAN	Tehran	35.69	51.39	86,990	911	1,132
IRAQ	Baghdad	33.31	44.36	42,043	3,149	913
ICELAND	Reykjavík	64.13	-21.82	364	220	77
ISRAEL	Tel Aviv	32.09	34.78	8,686	491	1,429
ITALY	Rome	41.90	12.50	59,640	9,540	8,613
JAMAICA	Kingston	18.04	-76.80	2,814	423	71
JERSEY	St. Helier	49.19	-2.11	103	18	15
JORDAN	Amman	31.96	35.95	10,817	536	212
JAPAN	Tokyo	35.71	139.73	125,543	3,188	2,545
KAZAKHSTAN	Astana	51.16	71.47	18,861	852	1,375
KENYA	Nairobi	-1.29	36.82	51,460	205	426
KYRGYZSTAN	Bishkek	42.87	74.57	6,372	851	673
CAMBODIA	Phnom Penh	11.54	104.89	16,296	661	310
KIRIBATI	Tarawa Atoll	1.45	172.97	125	1	1
ST. KITTS AND NEVIS	Basseterre	17.30	-62.72	48	111	5
SOUTH KOREA	Seoul	37.57	126.98	51,858	1,750	3,820
KUWAIT	Kuwait City	29.38	47.98	4,468	857	554
LAOS	Vientiane	17.98	102.63	7,266	479	392
LEBANON	Beirut	33.89	35.50	5,695	194	213
LIBERIA	Monrovia	6.29	-10.76	5,034	16	13
LIBYA	Tripoli	32.89	13.19	6,612	127	287
ST. LUCIA	Castries	14.01	-60.99	179	122	9
LIECHTENSTEIN	Vaduz	47.14	9.52	39	10	23
SRI LANKA	Sri Jayawardenepura Kotte	6.89	79.90	21,683	203	203
LESOTHO	Maseru	-29.36	27.51	2,240	114	11
LITHUANIA	Vilnius	54.69	25.28	2,836	615	549
LUXEMBOURG	Luxembourg	49.61	6.13	626	104	303
LATVIA	Riga	56.95	24.11	1,907	834	259
MACAU	Macau	22.17	113.55	670	3,941	154
ST. MARTIN	Marigot	18.07	-63.08	33	4	1
MOROCCO	Rabat	33.97	-6.85	36,489	1,311	314
MONACO	Monaco	43.74	7.42	37	36	29
MOLDOVA	Chisinau	47.01	28.86	3,095	17	37
MADAGASCAR	Antananarivo	-18.88	47.51	27,879	49	60
MALDIVES	Malé	4.18	73.51	511	170	23
MEXICO	Mexico City	19.43	-99.13	125,610	9,741	11,648
MARSHALL ISLANDS	Majuro	7.12	171.19	44	1	1
MACEDONIA (FYROM)	Skopje	42.00	21.43	2,114	76	50
MALI	Bamako	12.64	-8.00	20,887	22	71
MALTA	Valletta	35.90	14.51	510	352	109
MYANMAR (BURMA)	Naypyidaw	19.76	96.08	53,228	436	275
MONTENEGRO	Podgorica	42.43	19.26	630	251	22
MONGOLIA	Ulaanbaatar	47.89	106.91	3,266	64	55
NORTHERN MARIANA ISLANDS	Saipan	15.18	145.75	50	49	5
MOZAMBIQUE	Maputo	-25.89	32.61	30,721	203	68

MAURITANIA	Nouakchott	18.07	-15.96	4,441	14	33
MONTSERRAT	Plymouth	16.71	-62.22	5	2	0
MARTINIQUE	Fort-de-France	14.62	-61.06	371	92	10
MAURITIUS	Port Louis	-20.17	57.50	1,297	142	46
MALAWI	Lilongwe	-13.96	33.77	19,121	98	47
MALAYSIA	Kuala Lumpur	3.14	101.69	33,004	3,505	1,580
MAYOTTE	Mamoudzou	-12.78	45.23	321	1	2
NAMIBIA	Windhoek	-22.56	17.07	2,467	165	53
NEW CALEDONIA	Nouméa	-22.26	166.45	286	47	21
NIGER	Niamey	13.51	2.13	23,882	19	52
NORFOLK ISLAND	Kingston	-29.06	167.96	2	0	0
NIGERIA	Abuja	9.08	7.40	205,781	536	1,962
NICARAGUA	Managua	12.11	-86.24	6,710	146	151
NIUE	Alofi	-19.06	-169.92	2	1	0
NETHERLANDS	Amsterdam	52.37	4.90	17,402	2,013	2,343
NORWAY	Oslo	59.91	10.75	5,368	588	992
NEPAL	Kathmandu	27.72	85.32	28,999	120	126
NAURU	Yaren	-0.55	166.92	12	1	1
NEW ZEALAND	Wellington	-41.29	174.78	5,026	389	462
OMAN	Muscat	23.59	58.41	4,589	351	918
PAKISTAN	Islamabad	33.73	73.09	225,113	433	1,207
PANAMA	Panama City	9.10	-79.40	4,266	249	188
PERU	Lima	-12.05	-77.04	33,090	528	462
PHILIPPINES	Manila	14.60	120.98	111,288	826	1,415
PALAU	Ngerulmud	7.50	134.62	18	9	1
PAPUA NEW GUINEA	Port Moresby	-9.44	147.18	9,649	21	101
POLAND	Warsaw	52.23	21.01	38,478	8,852	5,671
PUERTO RICO	San Juan	18.47	-66.11	3,280	493	508
NORTH KOREA	Pyongyang	39.04	125.76	25,813	504	581
PORTUGAL	Lisbon	38.72	-9.14	10,298	1,728	508
PARAGUAY	Asuncion	-25.26	-57.58	6,573	437	555
PALESTINE	Ramallah	31.91	35.54	4,962	423	71
FRENCH POLYNESIA	Papeete	-17.55	-149.56	301	30	9
QATAR	Doha	25.29	51.53	2,829	214	743
RÉUNION	Saint-Denis	-20.88	55.45	954	60	5
ROMANIA	Bucharest	44.43	26.10	19,490	1,282	3,130
RUSSIA	Moscow	55.76	37.62	145,762	2,442	5,538
RWANDA	Kigali	-1.96	30.11	12,987	163	44
SAUDI ARABIA	Riyadh	24.75	46.90	36,230	2,029	4,027
SUDAN	Khartoum	15.50	32.56	43,828	839	134
SENEGAL	Dakar	14.76	-17.37	16,215	53	97
SINGAPORE	Singapore	1.28	103.85	5,894	1,912	1,821
SOUTH GEORGIA AND THE SOUTH SANDWICH ISLANDS	King Edward Point	-54.28	-36.49	1	0	0
SVALBARD AND JAN MAYEN	Longyearbyen	78.06	22.06	2	0	0
SOLOMON ISLANDS	Honiara	-9.45	159.97	683	3	7
SIERRA LEONE	Freetown	8.47	-13.23	8,140	7	17
EL SALVADOR	San Salvador	13.69	-89.22	6,281	264	305
SAN MARINO	San Marino	43.94	12.45	34	190	6
SOMALIA	Mogadishu	2.05	45.32	16,273	70	27
SAINT PIERRE AND MIQUELON	St. Pierre	46.78	-56.18	5	0	0
SERBIA	Belgrade	44.79	20.45	7,384	185	213
SOUTH SUDAN	Juba	4.86	31.57	10,545	45	51
SÃO TOMÉ AND PRÍNCIPE	São Tomé	0.33	6.73	216	3	2
SURINAME	Paramaribo	5.85	-55.20	604	7	16

SLOVAKIA	Bratislava	48.15	17.11	5,458	1,609	697
SLOVENIA	Ljubljana	46.06	14.51	2,116	470	921
SWEDEN	Stockholm	59.33	18.07	10,321	762	2,173
SWAZILAND Eswatini	Mbabane	-26.31	31.14	1,174	123	286
SEYCHELLES	Victoria	-4.62	55.45	105	43	11
SYRIA	Damascus	33.51	36.28	20,486	242	95
TURKS AND CAICOS ISLANDS	Cockburn Town	21.47	-71.14	44	160	6
CHAD	N'Djamena	12.13	15.06	16,379	8	46
TOGO	Lomé	6.17	1.23	8,342	88	36
THAILAND	Bangkok	13.76	100.50	71,389	3,992	1,426
TAJIKISTAN	Dushanbe	38.56	68.79	9,443	126	31
TOKELAU	Nukunonu	-9.20	-171.85	3	0	0
TURKMENISTAN	Ashgabat	37.96	58.33	6,204	229	169
TIMOR-LESTE	Dili	-8.56	125.56	1,290	8	8
TONGA	Nuku?alofa	-21.14	-175.20	105	9	2
TRINIDAD AND TOBAGO	Port of Spain	10.65	-61.50	1,514	48	103
TUNISIA	Tunis	36.81	10.18	12,106	943	390
TURKEY	Ankara	39.93	32.86	83,812	5,175	1,239
TUVALU	Funafuti	-8.52	179.20	11	0	1
TAIWAN	Taipei	25.03	121.57	23,801	1,186	2,125
TANZANIA	Dodoma	-6.16	35.75	60,772	153	267
UGANDA	Kampala	0.35	32.58	43,686	154	71
UKRAINE	Kiev	50.45	30.52	44,091	1,371	3,609
URUGUAY	Montevideo	-34.90	-56.16	3,428	348	289
UNITED STATES	Washington	38.91	-77.04	335,388	16,548	22,340
UZBEKISTAN	Tashkent	41.30	69.24	33,243	675	1,102
SAINT VINCENT AND THE GRENADINES	Kingstown	13.16	-61.22	105	40	4
VENEZUELA	Caracas	10.48	-66.90	28,629	316	1,008
BRITISH VIRGIN ISLANDS	Road Town	18.43	-64.62	31	90	1
U.S. VIRGIN ISLANDS	Charlotte Amalie	18.34	-64.93	101	207	18
VIETNAM	Hanoi	21.03	105.83	96,204	1,801	1,261
VANUATU	Port Vila	-17.73	168.33	308	26	5
WALLIS AND FUTUNA	Mata-Utu	-13.28	-176.18	11	0	1
SAMOA	Apia	-13.85	-171.75	213	18	10
KOSOVO	Pristina	42.66	21.17	1,800	434	184
YEMEN	Sana'a	15.37	44.19	31,927	2,392	86
SOUTH AFRICA	Pretoria	-25.75	28.23	58,466	1,480	1,629
ZAMBIA	Lusaka	-15.39	28.32	18,655	127	99
ZIMBABWE	Harare	-17.83	31.03	15,505	229	491

Table S4: Ranked AMR Travel Exposure Risk for Countries with Populations Greater than Five Million People

Country	Exposure Risk from Retuning Travellers LN(1+ERT)	Country	Exposure Risk from Inbound Visitors LN(1+ERV)	Country	AMR Burden of Travel LN(1+ERT+ERV)
HONG KONG	1.80	HONG KONG	1.04	HONG KONG	2.06
SWEDEN	0.59	DENMARK	0.83	DENMARK	0.97
FINLAND	0.53	HUNGARY	0.59	NEW ZEALAND	0.81
NORWAY	0.52	AUSTRIA	0.52	HUNGARY	0.78
NEW ZEALAND	0.52	FRANCE	0.50	NORWAY	0.76
ISRAEL	0.47	NEW ZEALAND	0.45	SWEDEN	0.74
SWITZERLAND	0.41	CZECH REPUBLIC	0.40	AUSTRIA	0.68
HUNGARY	0.32	GREECE	0.37	FINLAND	0.65
BELGIUM	0.31	NORWAY	0.37	ISRAEL	0.59
DENMARK	0.30	SINGAPORE	0.32	FRANCE	0.58
SINGAPORE	0.30	SPAIN	0.30	SWITZERLAND	0.57
UNITED KINGDOM	0.27	POLAND	0.27	SINGAPORE	0.54
NETHERLANDS	0.26	SWEDEN	0.27	CZECH REPUBLIC	0.47
AUSTRIA	0.26	UNITED ARAB EMIRATES	0.25	NETHERLANDS	0.46
UNITED ARAB EMIRATES	0.24	NETHERLANDS	0.25	GREECE	0.45
GERMANY	0.22	SWITZERLAND	0.23	UNITED ARAB EMIRATES	0.44
CANADA	0.22	PORTUGAL	0.22	BELGIUM	0.41
PARAGUAY	0.20	SLOVAKIA	0.20	POLAND	0.41
ARGENTINA	0.19	BULGARIA	0.20	SPAIN	0.35
AUSTRALIA	0.18	FINLAND	0.19	UNITED KINGDOM	0.35
POLAND	0.17	ISRAEL	0.19	PARAGUAY	0.34
BULGARIA	0.14	PARAGUAY	0.16	CANADA	0.32
TAIWAN	0.13	MALAYSIA	0.15	BULGARIA	0.31
FRANCE	0.13	ITALY	0.14	GERMANY	0.29
ROMANIA	0.12	CANADA	0.13	ARGENTINA	0.28
GREECE	0.12	BELGIUM	0.13	PORTUGAL	0.28
ITALY	0.11	AUSTRALIA	0.11	AUSTRALIA	0.27
SAUDI ARABIA	0.10	ARGENTINA	0.11	SLOVAKIA	0.27
CZECH REPUBLIC	0.09	UNITED KINGDOM	0.11	ITALY	0.23
UNITED STATES	0.09	BELARUS	0.10	MALAYSIA	0.21
BELARUS	0.08	TUNISIA	0.10	TAIWAN	0.20
SLOVAKIA	0.08	KYRGYZSTAN	0.10	BELARUS	0.17
SOUTH KOREA	0.07	GERMANY	0.08	ROMANIA	0.17
KYRGYZSTAN	0.07	TAIWAN	0.07	KYRGYZSTAN	0.17
SPAIN	0.07	UNITED STATES	0.07	UNITED STATES	0.15
MALAYSIA	0.07	LAOS	0.06	SAUDI ARABIA	0.14
PORTUGAL	0.06	ROMANIA	0.06	TUNISIA	0.14

MEXICO	0.06	DOMINICAN REPUBLIC	0.06	MEXICO	0.11
UKRAINE	0.06	YEMEN	0.05	LAOS	0.11
AZERBAIJAN	0.05	MEXICO	0.05	SOUTH KOREA	0.11
LAOS	0.05	COSTA RICA	0.05	SOUTH AFRICA	0.08
KAZAKHSTAN	0.05	SAUDI ARABIA	0.05	SERBIA	0.08
VENEZUELA	0.05	MOROCCO	0.05	KAZAKHSTAN	0.08
SOUTH AFRICA	0.04	JORDAN	0.05	UKRAINE	0.08
SERBIA	0.04	THAILAND	0.04	COSTA RICA	0.08
TUNISIA	0.04	IRAQ	0.04	AZERBAIJAN	0.08
EL SALVADOR	0.04	SOUTH AFRICA	0.04	CHILE	0.07
CHILE	0.04	SERBIA	0.04	EL SALVADOR	0.07
BRAZIL	0.03	TURKEY	0.04	JORDAN	0.06
LIBYA	0.03	CHILE	0.04	DOMINICAN REPUBLIC	0.06
RUSSIA	0.03	SOUTH KOREA	0.04	VENEZUELA	0.06
COSTA RICA	0.03	JAPAN	0.03	JAPAN	0.06
LEBANON	0.03	EL SALVADOR	0.03	YEMEN	0.06
JAPAN	0.03	KAZAKHSTAN	0.03	THAILAND	0.06
UZBEKISTAN	0.02	TURKMENISTA N	0.03	MOROCCO	0.06
TURKMENISTAN	0.02	LEBANON	0.02	IRAQ	0.05
JORDAN	0.02	UKRAINE	0.02	LEBANON	0.05
ZIMBABWE	0.02	AZERBAIJAN	0.02	TURKEY	0.05
NORTH KOREA	0.02	CUBA	0.02	TURKMENISTAN	0.05
NICARAGUA	0.02	VIETNAM	0.02	LIBYA	0.04
PAPUA NEW GUINEA	0.02	HONDURAS	0.02	RUSSIA	0.04
VIETNAM	0.02	CAMBODIA	0.02	UZBEKISTAN	0.04
THAILAND	0.01	NICARAGUA	0.02	BRAZIL	0.04
ECUADOR	0.01	NORTH KOREA	0.02	VIETNAM	0.04
GUATEMALA	0.01	UZBEKISTAN	0.02	NORTH KOREA	0.03
BOLIVIA	0.01	VENEZUELA	0.01	NICARAGUA	0.03
IRAQ	0.01	LIBYA	0.01	HONDURAS	0.03
COLOMBIA	0.01	ECUADOR	0.01	CUBA	0.03
PHILIPPINES	0.01	RUSSIA	0.01	CAMBODIA	0.03
MOROCCO	0.01	GUATEMALA	0.01	ECUADOR	0.03
HONDURAS	0.01	RWANDA	0.01	ZIMBABWE	0.03
CAMBODIA	0.01	SYRIA	0.01	GUATEMALA	0.03
TURKEY	0.01	BURUNDI	0.01	BOLIVIA	0.02
ALGERIA	0.01	SUDAN	0.01	PAPUA NEW GUINEA	0.02
CHINA	0.01	TAJIKISTAN	0.01	COLOMBIA	0.02
IRAN	0.01	BOLIVIA	0.01	PHILIPPINES	0.02
PERU	0.01	ZIMBABWE	0.01	SYRIA	0.02
EGYPT	0.01	MOZAMBIQUE	0.01	CHINA	0.02
DOMINICAN REPUBLIC	0.01	COLOMBIA	0.01	IRAN	0.01
CUBA	0.01	PERU	0.01	PERU	0.01
SRI LANKA	0.01	CHINA	0.01	RWANDA	0.01

ANGOLA	0.00	IRAN	0.01	EGYPT	0.01
NIGERIA	0.00	TOGO	0.01	BURUNDI	0.01
SYRIA	0.00	EGYPT	0.01	TAJIKISTAN	0.01
HAITI	0.00	PHILIPPINES	0.01	SUDAN	0.01
GHANA	0.00	SRI LANKA	0.01	ALGERIA	0.01
BANGLADESH	0.00	HAITI	0.01	SRI LANKA	0.01
CÔTE D'IVOIRE	0.00	MYANMAR (BURMA)	0.00	MOZAMBIQUE	0.01
PAKISTAN	0.00	MALAWI	0.00	HAITI	0.01
SENEGAL	0.00	PAPUA NEW GUINEA	0.00	TOGO	0.01
CONGO (REPUBLIC)	0.00	ZAMBIA	0.00	CÔTE D'IVOIRE	0.01
INDONESIA	0.00	CÔTE D'IVOIRE	0.00	MYANMAR (BURMA)	0.01
CAMEROON	0.00	LIBERIA	0.00	ZAMBIA	0.01
RWANDA	0.00	INDONESIA	0.00	NIGERIA	0.01
KENYA	0.00	ALGERIA	0.00	INDONESIA	0.01
ZAMBIA	0.00	BRAZIL	0.00	GHANA	0.01
MOZAMBIQUE	0.00	NEPAL	0.00	LIBERIA	0.01
NEPAL	0.00	CAMEROON	0.00	MALAWI	0.01
TANZANIA	0.00	UGANDA	0.00	ANGOLA	0.01
MYANMAR (BURMA)	0.00	SOUTH SUDAN	0.00	CAMEROON	0.01
TOGO	0.00	MADAGASCAR	0.00	SENEGAL	0.01
TAJIKISTAN	0.00	SENEGAL	0.00	NEPAL	0.00
LIBERIA	0.00	GHANA	0.00	PAKISTAN	0.00
BENIN	0.00	SOMALIA	0.00	SOUTH SUDAN	0.00
MADAGASCAR	0.00	GUINEA	0.00	MADAGASCAR	0.00
MALI	0.00	BENIN	0.00	KENYA	0.00
BURUNDI	0.00	TANZANIA	0.00	TANZANIA	0.00
SOUTH SUDAN	0.00	NIGERIA	0.00	BENIN	0.00
YEMEN	0.00	KENYA	0.00	BANGLADESH	0.00
GUINEA	0.00	PAKISTAN	0.00	CONGO (REPUBLIC)	0.00
MALAWI	0.00	AFGHANISTAN	0.00	GUINEA	0.00
SUDAN	0.00	CONGO (DRC)	0.00	MALI	0.00
ETHIOPIA	0.00	CENTRAL AFRICAN REPUBLIC	0.00	UGANDA	0.00
INDIA	0.00	MALI	0.00	SOMALIA	0.00
CHAD	0.00	INDIA	0.00	CONGO (DRC)	0.00
CONGO (DRC)	0.00	CONGO (REPUBLIC)	0.00	AFGHANISTAN	0.00
BURKINA FASO	0.00	SIERRA LEONE	0.00	INDIA	0.00
SIERRA LEONE	0.00	ANGOLA	0.00	CENTRAL AFRICAN REPUBLIC	0.00
AFGHANISTAN	0.00	NIGER	0.00	ETHIOPIA	0.00
CENTRAL AFRICAN REPUBLIC	0.00	ETHIOPIA	0.00	SIERRA LEONE	0.00
NIGER	0.00	BURKINA FASO	0.00	BURKINA FASO	0.00
UGANDA	0.00	CHAD	0.00	CHAD	0.00
SOMALIA	0.00	BANGLADESH	0.00	NIGER	0.00

Supplement References

1. Zipf, G. K. (1946). The P1P2/D hypothesis: On the intercity movement of persons. American Sociological Review, 11(6), 677–686
2. Tinbergen, J. (1962). Shaping the world economy: Suggestions for an International Economic Policy. The Twentieth Century Fund.
3. Anderson, J. E. (1979). A theoretical foundation for the gravity equation. American Economic Review, 69, 106–16
4. Anderson, J.E. The Gravity Model, Working Paper 16576 http://www.nber.org/papers/w16576 NATIONAL BUREAU OF ECONOMIC RESEARCH 1050 Massachusetts Avenue Cambridge, MA 02138 December 2010
5. Wilson, A.G. A statistical theory of spatial distribution models. Transportation Research 1967 1,3 253-269
6. Mathisen, Terje Andreas Empirical Evidence on the Relationship between Fare and Travel Distance International Journal of Transport Economics Volume: 42 Issue Number: 1 2015
7. McKercher, Bob, Andrew Chan, Celia Lam The Impact of Distance on International Tourist Movements Journal of Travel Research Volume 47 Number 2 November 2008, 08-224
8. Urbonavicius, S., R. Andruliene2, K. Adomaviciute, D. Ozretic-Dosen, The role of travelling distances in tourism: different planning motives Bulletin of Geography. Socio-economic Series, No. 60 2023 : 145-156
9. Jørgensen, F. and John Preston, The Relationship Between Fare and Travel Distance Journal of Transport Economics and Policy, Volume 41, Part 3, September 2007, pp. 451–468
10. Nadal J.R., María, Gallego S. Gravity models for tourism demand modeling: Empirical review and outlook J Economic Surveys 2022;36:1358–1409.
